# Ultrasound priming gated by solid tumor hallmarks to guide CAR-T therapy

**DOI:** 10.1126/sciadv.aed0666

**Published:** 2026-06-10

**Authors:** Tianze Guo, Ziyue Zhu, Yunjia Qu, Xi Yu, Linshan Zhu, Yuxuan Wang, Kunshu Liu, Jiaxin Cui, Xue Dong, Chi-Woo Yoon, Cem Yigit Kilic, Peixiang He, Shuangquan Gou, Yushun Zeng, Priyankan Datta, Qifa Zhou, Hongquan Xu, Ishwar K. Puri, Longwei Liu, Keyue Shen, Yingxiao Wang

**Affiliations:** ^1^ Alfred E. Mann Department of Biomedical Engineering, University of Southern California, Los Angeles, CA, USA.; ^2^ Shu Chien-Gene Lay Department of Bioengineering, Institute of Engineering in Medicine, University of California, San Diego, CA, USA.; ^3^ Department of Ophthalmology, Roski Eye Institute, Keck School of Medicine, University of Southern California, Los Angeles, CA, USA.; ^4^ Department of Aerospace and Mechanical Engineering, University of Southern California, Los Angeles, CA, USA.; ^5^ Department of Statistics and Data Science, University of California, Los Angeles, Los Angeles, CA, USA.

## Abstract

CAR-T therapy is highly effective in hematologic malignancies but remains challenging in solid tumors. To address this challenge, here, we developed SHIFTERS (solid-tumor hallmark inducible, focused-ultrasound triggered enhanced reprogramming system), an AND-gated circuit that rewires tumor hallmarks into the clinically validated antigen CD19 under focused-ultrasound (FUS) control. SHIFTERS uses a split Gal4-VP64 transcription factor with one module driven by a hypoxia-responsive promoter and the other by a FUS-inducible heat-shock promoter, restricting CD19 induction to sonicated, hallmark-positive regions. This design enables robust, sustained local CD19 expression to activate and train CD19 synNotch CAR-T cells. In 3D spheroids and in vivo models, hypoxia-gated SHIFTERS combined with synNotch CAR-T cells achieved strong tumor suppression, supporting FUS-guided, localized activation. SHIFTERS is modular and can be adapted to tumor-type–specific promoters such as enAFP in hepatocellular carcinoma to enable both spatial and cell type–specific targeting. Together, SHIFTERS provides a modular, tumor-constrained, ultrasound-controllable platform to improve the precision and efficacy of CAR-T therapy for solid tumors.

## Teaser

Ultrasound plus tumor cues safely prime CAR-T cells on site, enabling precise, durable attacks on solid tumors.

## INTRODUCTION

Chimeric antigen receptor T cell (CAR-T) therapy has revolutionized cancer treatment. Despite notable advancements in blood cancers, expanding the application of CAR-T cell therapy to solid tumors remains challenging, requiring considerable efforts to enhance its safety and efficacy (*1*). On one hand, safety concerns, primarily stemming from on-target off-tumor toxicity, complicate clinical use (*2*, *3*). On the other hand, solid tumors comprise heterogeneous cell populations with limited tumor-specific antigens, undermining the efficacy of CAR-T cell therapy. Furthermore, even when these antigens are initially present, their expression can be down-regulated or lost over time, allowing tumor cells to evade CAR-T cell–mediated killing, an effect known as “antigen escape” (*4*). To address these challenges, current strategies include using multivalent CARs, which enable CAR-T cells to simultaneously recognize multiple antigens, and using switchable universal CAR-T platforms that offer adaptable antigen targeting (*5*–*8*). Other approaches are employed to introduce synthetic orthogonal antigens into tumor cells that are recognizable by CAR-T cells but absent from normal tissues, which minimizes off-tumor safety (*9*, *10*).

Tumors have unique environmental features, such as hypoxia and distinct transcriptional profiles, that distinguish them from normal tissues (*11*–*13*). For hypoxia, up to 90% of solid tumors exhibit inadequate oxygen levels resulting from rapid growth and irregular vasculature that cannot meet their elevated oxygen demands (*13*–*16*). In response, tumor cells activate the hypoxia signaling pathway via HIF1-α nuclear localization, forming complexes with HIF1 factors to promote transcription of hypoxia-regulated genes (*17*–*20*). This signaling pathway has been used to drive gene expression in the tumor region through the hypoxia response element (HRE) (*21*–*25*). However, while solid tumors are predominantly hypoxic, certain nontumor tissues, such as the brain, liver, bone marrow, and rectal mucosa (*26*, *27*), can also experience low oxygen levels that initiate off-tumor expression of target genes and unintended toxicities in these organs. Therefore, further refinement of hypoxia-synthesized therapy is essential to enhance both its selectivity and safety. In parallel, tumor cells often display altered transcriptional regulations due to oncogenic mutations and disrupted signaling pathways, generating molecular signatures whose associated promoters can serve as tumor-specific regulatory elements (*28*–*30*). Recent advances in synthetic biology have enabled the design of synthetic circuits that integrate such promoters to restrict the expression of therapeutic payloads to malignant cells (*31*–*33*). Noninvasive stimulation platforms such as ultrasound-based sonogenetics can enhance spatiotemporal precision (*34*, *35*), as these promoters are rarely strictly specific to the tumor cells. Owing to its capability to penetrate deeply and induce localized hyperthermia in biological tissues without the need for intermediate cofactors (*36*–*38*), focused ultrasound (FUS) has been used to achieve spatiotemporal control of heat-sensitive transgene expression in vivo at mildly elevated temperatures (43°C) through activation of the innate heat shock response combined with a heat-shock promoter (*39*–*42*).

We developed solid-tumor hallmark inducible, focused-ultrasound triggered enhanced reprogramming system (SHIFTERS), a platform designed to enable targeted and durable expression of clinically validated antigens in solid tumors, thereby facilitating effective and safe synNotch CAR-T cell therapy. By gating FUS with hypoxia or tumor-specific promoters representing tumor hallmarks, the SHIFTERS platform provides a tailored solution that can confine and enhance clinical antigen expression to the tumor region over a therapeutically relevant and sustained timeframe.

## RESULTS

### Developing SHIFTERS with controlled and enhanced production of CD19 under hypoxia and FUS

**Fig. 1.**
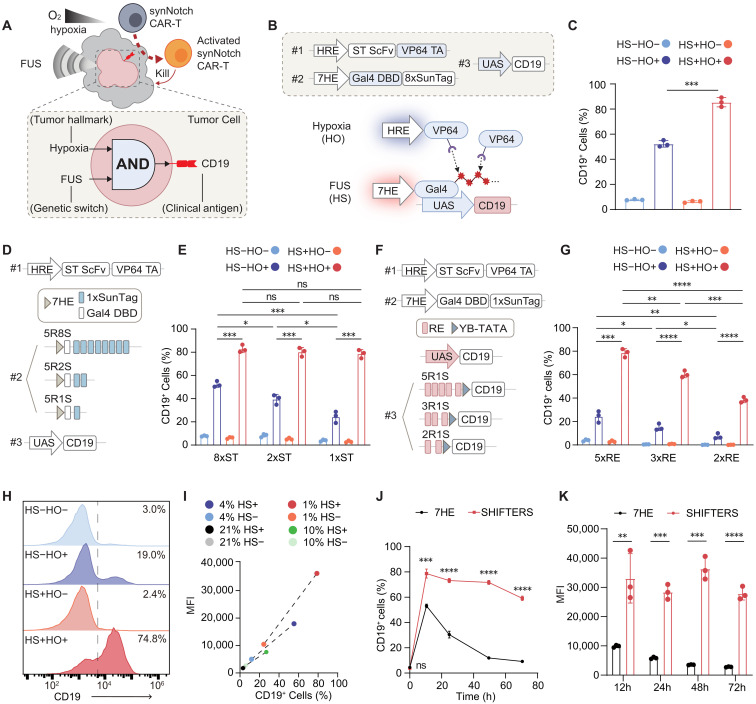
Developing SHIFTERS with controlled and enhanced production of CD19 under hypoxia and FUS. (**A**) Schematic of tumor-specific synNotch CAR-T activation. In this AND-gated system, focused ultrasound (FUS) induces CD19 expression selectively under hypoxia, enabling anti-CD19 synNotch CAR-T cells to activate EGFR CAR expression and kill EGFR+ tumor cells. (**B**) Gene constructs of the system shown in (A). (**C**) Flow cytometry data for CD19 induction across four conditions (HS, heat shock; HO, hypoxia). (**D**) Schematic of SunTag optimization (#2). (**E**) Percentage of CD19^+^ cells for different SunTag (ST) configurations with or without hypoxia and heat shock. Signal/noise ratio: 1.58 (8xST), 2.03 (2xST), and 3.23 (1xST). (**F**) Schematic of UAS optimization. (**G**) Flow cytometry analysis on percentage of CD19^+^ cells for different UAS configurations. Signal/noise ratio: 3.23 (5xRE), 4.01 (3xRE), and 5.23 (2xRE). (**H**) Representative histograms show CD19 expression across all four conditions for the SHIFTERS configuration. (**I**) Evaluation of CD19 expression under varying oxygen levels: normoxia, 21% O_2_; physioxia, 10 and 4% O_2_ (highest and lowest physioxia); and hypoxia: 1% O_2_. Each dot represents the average among the samples in each group (*n* = 3). Notably, under physiologic oxygen tensions (10 to 4%), CD19 remained near baseline, with low CD19^+^ fractions and low MFI compared with 1% O_2_, indicating that basal induction is minimal in physioxia and becomes prominent primarily under tumor-like hypoxia. (**J**) Comparison of CD19^+^ cell percentages over time between SHIFTERS under HO+HS+ and 7HE-driven CD19 under HS only; *t* = 0 represents no hypoxia or heat shock. (**K**) Mean fluorescence intensity (MFI) of CD19 shown in (J). Statistical significance: ns, not significant; *P < 0.05, **P < 0.01, ***P < 0.001, and ****P < 0.0001. All data are presented as mean ± SD (*n* = 3). Two tailed Student’s *t* tests are applied for all the statistical analysis.

SHIFTERS is an AND-gate design that uses a split Gal4-VP64 transcription activator, with Gal4 capable of binding to the DNA upstream activating sequence (UAS) (*43*) and VP64 initiating transcription (*44*). Gal4-VP64 has been used in various applications to amplify transcriptional activity by rewiring weak transcriptional signals to the Gal4-UAS signaling pathway (*28*, *45*). To engineer an AND-gate system which enhances controllability and sustains activation after induction, we designed a split Gal4-VP64–based system controlled by both hypoxia and FUS (or FUS-induced heat shock). Specifically, we used hypoxia-responsive promoter HRE to drive the expression of VP64 transcriptional activator (VP64 TA) along with a single-chain variable fragment binder specific to SunTag (ST ScFv). Heat shock promoter 7HE (*41*) was applied to express the Gal4 DNA binding domain (Gal4 DBD) fused to eight tandem SunTag repeats (8xSunTag), ensuring robust binding and signal amplification (*46*, *47*). Previous studies demonstrated that expressing either the Gal4 or the VP64 alone does not initiate Gal4UAS-driven transcription (*48*, *49*). Consequently, only when both hypoxia and FUS conditions are met, Gal4UAS drives transcription of CD19 (Fig. 1, A and B). CD19 is a clinically validated antigen that can be expressed on solid tumor cells to enable targeting by CAR-T therapies, overcoming the lack of suitable tumor-specific markers in solid tumors (*50*, *51*). We genetically engineered and examined U251-MG (U251) glioblastoma cells under four conditions: with or without hypoxia (1% O_2_) and with or without heat shock (43°C, 15 min) (*52*, *53*). A significant increase in CD19^+^ cells under the presence of both hypoxia and heat shock was observed (Fig. 1C). However, we also detected CD19 expression under hypoxia alone. Since ScFv-VP64 alone cannot initiate UAS-mediated transcription, we hypothesized that the basal leakage of the 7HE promoter produced low levels of Gal4-Suntag even without heat shock and resulted in UAS transcription under hypoxia-only condition. To mitigate this problem, we reduced the number of SunTag repeats (Fig. 1D, #2), which successfully diminished background CD19 production under hypoxia-only conditions (Fig. 1E). Consequently, the signal/noise ratio of SHIFTERS substantially improved with 1xSunTags (Fig. 1E). Notably, despite fewer SunTag repeats, the system still retained high CD19 expression induction when both hypoxia and heat shock were present (Fig. 1E), suggesting the robustness of SHIFTERS.

The standard UAS contains five tandem UAS response elements (REs) and a minimal yb-TATA promoter, where the number of RE determines the strength of downstream transcription (*54*, *55*). Therefore, we truncated the UAS promoter to 3× and 2× RE repeats to further minimize CD19 background and examined the resulting effects (Fig. 1F, #3). Reducing the number of RE successfully lowered background activation and increased the signal/noise ratio of SHIFTERS. However, this improvement came at the cost of significantly reduced CD19 expression under both hypoxia and heat shock (Fig. 1G). Since synNotch only recognizes CD19 at relatively high levels (*56*), a weak basal CD19 expression may be tolerable without triggering notable nonspecific synNotch CAR T activation. We hence selected 5R1S as the final product (Fig. 1H). We quantified CD19 expressions under several oxygen levels: 21% for normoxia, 10% and 4% for the highest and lowest physioxia (*26*, *27*), and 1% for tumor-like hypoxia. As the oxygen level decreased, the CD19^+^ percentage and level increased (Fig. 1I), confirming that the system is indeed responsive to lower oxygen concentrations. Furthermore, compared to the transient CD19 expression directly driven by heat shock (7HE-CD19), SHIFTERS turned on an enhanced and sustained CD19 production (Fig. 1, J and K), potentially could lead to enhanced synNotch CAR-T cell activation. Nevertheless, CD19 expression returned to basal levels by day 6 post–heat shock stimulation, demonstrating that SHIFTERS retains reversibility despite its prolonged expression kinetics (fig. S1).

### SHIFTERS induces CD19 expression in tumors to form “training center” that activates synNotch CAR-T cells for therapeutics

**Fig. 2.**
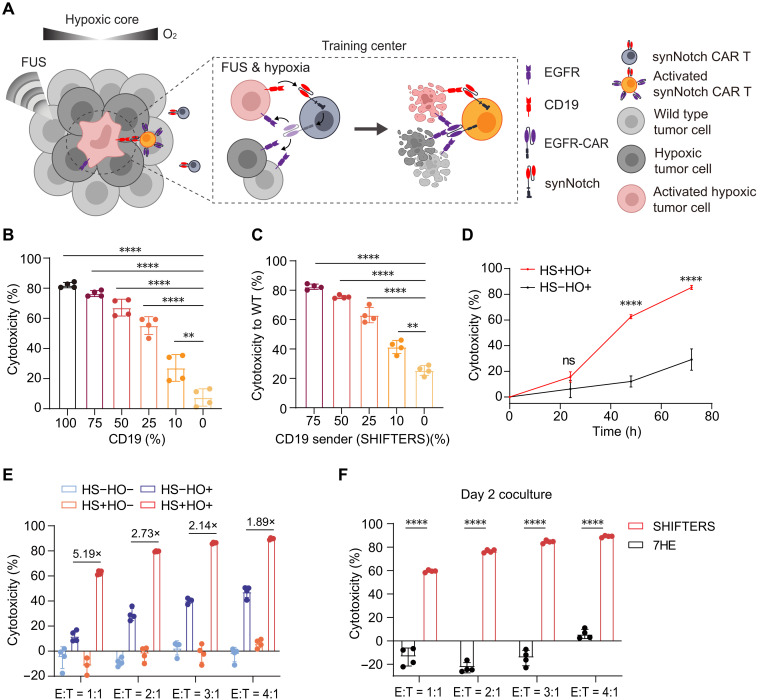
SHIFTERS induces CD19 expression in tumors to form training center that activates synNotch CAR-T cells for therapeutics. (**A**) Schematic illustration of the tumor training center concept, where a subset of tumor cells with CD19 expression under both hypoxia and FUS stimulation induces CD19 synNotch T cells to produce EGFR CARs, enabling the killing of both engineered CD19^+^EGFR^+^ and nonengineered CD19^−^EGFR^+^ tumor cells. (**B**) Cytotoxicity analysis of CD19 synNotch EGFR CAR-T cells against U251 cells with varying fractions of constitutive CD19^+^ cells. (**C**) Cytotoxicity analysis of CD19 synNotch EGFR CAR-T cells exclusively against wild-type U251 cells, with varying fractions of SHIFTERS sender cells producing CD19 upon AND-gate stimulation. (**D**) Time course of cytotoxicity of CD19 synNotch EGFR CAR-T cells killing tumor cells in heat-shock plus hypoxia (HS+ HO+) or non-heat-shock hypoxia-only (HS− HO+) conditions. (**E**) Cytotoxicity results at different E:T ratios of CD19 synNotch EGFR CAR-T cells with tumor cells under all four combinations of heat shock (HS) and hypoxia (HO), examined 48 hours after coculture. (**F**) Comparison of cytotoxicity elicited by SHIFTERS and 7HE constructs at various E:T ratios with CD19 synNotch EGFR CAR-T cells on 48 hours after coculture on day 2. SHIFTERS U251 was cultured constantly under hypoxia conditions whereas 7HE group stayed at normoxic throughout coculture. Statistical significance: ns, not significant; **P < 0.01 and ****P < 0.0001. All data are presented as mean ± SD (*n* = 4). Two tailed Student’s *t* tests are applied for statistical analysis.

SHIFTERS can be applied to induce CD19 expression in a subpopulation of U251 cancer cells exposed to hypoxia and FUS, which can form “training centers” to activate and enable CD19 synNotch T cells to produce epidermal growth factor receptor (EGFR)–CARs, capable of targeting and eliminating the entire population of U251 cells overexpressing EGFR (Fig. 2A). To examine the concept of this “training center” approach, we first introduced U251 cells with constitutive CD19 and mixed CD19^+^ and CD19^−^ U251 cells with various ratios. We then added CD19 synNotch CAR-T cells in a 1:1 ratio for coculture and examined the killing effect. With 25% CD19^+^, more than half of the cancer cell population was cleared, demonstrating that a small subset of “trainer” cells can initiate substantial tumor cell killing (Fig. 2B). Next, we replaced the constitutive CD19^+^ U251 cells with SHIFTERS U251 to evaluate the effect of AND-gate-based training centers. The SHIFTERS engineered U251 cells were mixed with the wild-type U251 cells and cocultured with CD19 synNotch CAR-T cells for 3 days under hypoxia after heat shock. To precisely validate the “training center” function, we only measured the bystander cytotoxicity exclusively from the mixed wild-type U251 population. Increasing the proportion of SHIFTERS cells significantly enhanced the bystander killing of wild-type U251 cells. Even at a 10% SHIFTERS cells, >40% cytotoxicity can be detected of the wild-type U251 cells (Fig. 2C). Wild-type U251 cells cocultured with plain T cells or constitutive EGFR CAR-T cells both showed no significant differences in cytotoxicity between hypoxia (1% O_2_) and normoxia (21% O_2_) (fig. S2), confirming that hypoxia itself does not affect cancer cell death. These results underscore that the AND-gated SHIFTERS system also effectively “trains” synNotch CAR T cells guided by the remote FUS stimulation to eliminate the whole population of EGFR-expressing tumor cells, both engineered and nonengineered, highlighting the potential of this approach for targeted and controlled tumor eradication.

To further assess the specificity and safety of SHIFTERS-driven CAR activation, we monitored the cytotoxicity time course of synNotch CAR-T cells against activated SHIFTERS tumor cells (1:1 ratio). In parallel, we included a non-heat-shock control to determine whether the basal leakage of CD19 expression under hypoxia alone could trigger significant synNotch CAR-T cell killing. Under both hypoxia and heat shock, CAR-T killing rose as time progressed and reached 80% at day 3, while the non-heat-shock control increased only slightly, confirming that the hypoxia-only induced CD19 leakage was insufficient for strong CD19 synNotch EGFR CAR-T cell responses (Fig. 2D). We then tested all four conditions (heat-shock versus no heat-shock and hypoxia versus no hypoxia) in coculture with CD19 synNotch CAR-T cells at varying effector-to-target (E:T) ratios. At an E:T ratio of 1:1, only the fully activated condition (heat-shock plus hypoxia) showed robust cytotoxicity. As the E:T ratio increased, nonspecific cytotoxicity under the hypoxia-only condition also increased, reducing the relative specificity advantage of the AND-gated design. Thus, an E:T ratio of 1:1 was confirmed to achieve strong cytotoxicity and specificity (Fig. 2E). We further found that SHIFTERS provided a longer therapeutic window for SynNotch CAR-T activation than 7HE-CD19, driving stronger cytotoxicity both at immediate coculture after stimulation (peak CD19 with 7HE) and with delayed coculture (Fig. 2F and fig. S3). This advantage is consistent with the antigen induction kinetics in Fig. 1 (J and K), reflecting SHIFTERS’ durable CD19 expression that prolongs the window for CAR-T activation.

### SHIFTERS together with synNotch CAR-T cells showed effective tumor suppression in a spheroid model

**Fig. 3.**
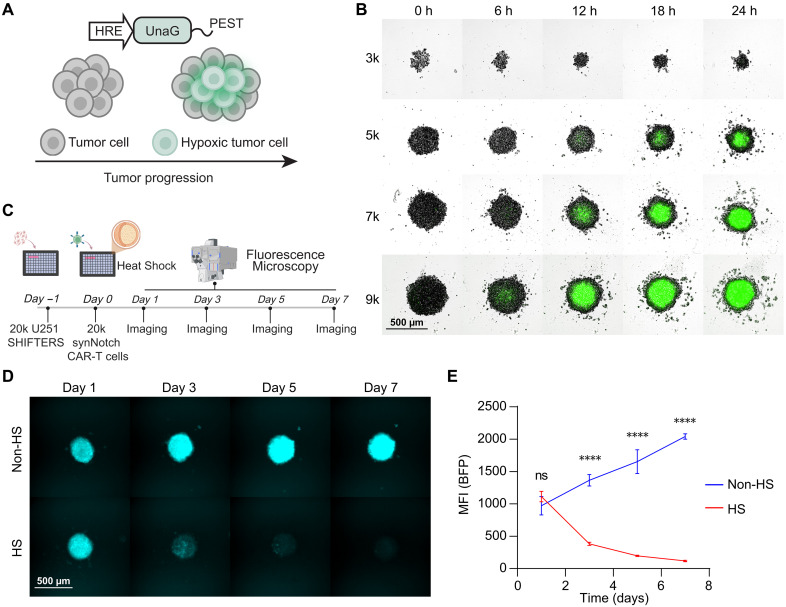
SHIFTERS together with synNotch CAR-T cells showed effective tumor suppression in a spheroid model. (**A**) Schematic of the hypoxia biosensor construct: a hypoxia response element (HRE) promoter driving UnaG with a PEST degron, enabling green fluorescence under hypoxic conditions. (**B**) Time-lapse images of spheroids formed from 3000, 5000, 7000, and 9000 U251 cells, taken from IncuCyte culture. UnaG expression is obtained with a standard GFP filter, bright-field images and fluorescence images were stacked using ImageJ, scale bar is in lower left referencing 500 μm length, the scales are consistent in all subpanels of this figure. (**C**) Experimental timeline for spheroid formation and imaging. U251 SHIFTERS cells (20,000) are plated 1 day before spheroid formation, then combined with CD19 synNotch EGFR CAR-T cells (20,000) on day 0. Heat shock (HS) treatment is applied as indicated, and spheroids are imaged at 24, 72, 120, and 168 (days 1, 3, 5, and 7). Created in BioRender. Guo, T. (2026) https://BioRender.com/xgy1rrs. (**D**) Representative fluorescence images of U251 spheroids expressing BFP at days 1, 3, 5, and 7, comparison between non-HS and HS groups. Merged-channel images (BFP and bright-field) were generated by ImageJ and brightness/intensity thresholds were normalized. The scale bar in the lower left referencing 500 μm length; the scales are consistent in all subpanels of this figure. (**E**) Mean fluorescence intensity (MFI) of constitutive marker BFP in spheroids over time under non-HS and HS conditions, data analyzed using microscopy images using ImageJ. Data are presented as mean ± SD. Two tailed Student’s t tests are applied for statistical analysis (*n* = 4). Statistical significance: ns, not significant; ****P < 0.0001.

We next examined the performance of SHIFTERS in a three-dimensional (3D) spheroid tumor model (*57*, *58*). To evaluate the development of hypoxia in the 3D cultured spheroid model, we first applied an established HRE-based biosensor to visualize the activation of HIF signaling (*24*, *25*) (Fig. 3A). As the tumor spheroid grew, green UnaG fluorescence signals gradually increased, verifying the formation of a hypoxic microenvironment, with the intensity proportional to the starting cell number and the spheroid growth duration (Fig. 3B and movie S1). Engineered SHIFTERS U251 spheroids with or without heat shock were then cocultured with CD19 synNotch CAR-T cells and the spheroid destruction was monitored using a constitutive blue fluorescence protein (BFP) marker (Fig. 3C). Heat-shocked spheroids showed a rapid decline in BFP signal, reflecting effective tumor clearance, while non-heat-shocked spheroids accumulated BFP continuously, indicating tumor growth (Fig. 3, D and E). Complementary spheroid size quantification confirmed this observation (fig. S6A). This stark contrast underscores the strength of SHIFTERS combined with CD19 synNotch CAR-T in eliminating hypoxic tumors under heat shock stimulation, while nonspecific background killing remained insignificant in the non-heat-shocked controls. To further validate this observation, spheroids seeded without T cells were exposed to heat shock or control conditions. Both heat-shocked and non-heat-shocked groups showed similar growth curves, indicating that heat shock alone does not influence spheroid growth (fig. S4A). Similar results were observed following plain T cell treatment, confirming the specificity of the CD19 synNotch CAR-T treatment (fig. S4B). We further performed confocal imaging and observed infiltration of CD19 synNotch CAR-T cells into activated SHIFTERS U251 spheroids, supporting the engagement with tumor cells within hypoxic niches (fig. S5).

### SHIFTERS demonstrates modular promoter adaptability across diverse tumor contexts

**Fig. 4.**
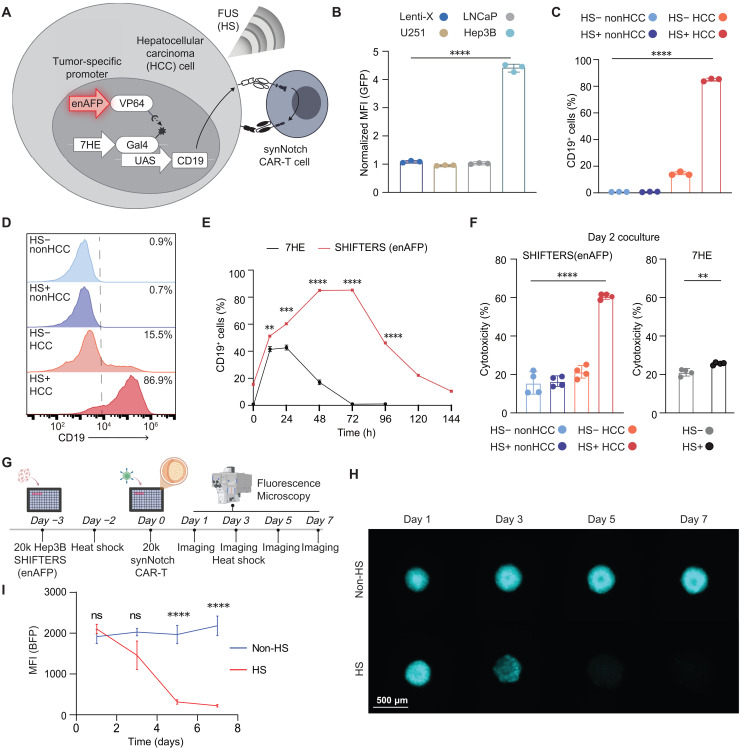
SHIFTERS demonstrates modular promoter adaptability across diverse tumor contexts. (**A**) Schematic of tumor-specific synNotch activation by SHIFTERS using a liver tumor-specific promoter. (**B**) Flow cytometry analysis enAFP-driven GFP expression (normalized to nontransduced population) across four cell lines: Lenti-X, LNCaP, and U251 (nonHCC), and Hep3B (HCC). (**C**) Flow cytometry of CD19 expression from the construct in (A) under four conditions: with or without heat shock (HS), in HCC or non-HCC cells. (**D**) Representative flow cytometry histograms showing CD19 expression levels across the four tested conditions. (**E**) Temporal comparison of CD19 expression in enAFP-based SHIFTERS (HCC, HS+) versus a 7HE-only construct (*n* = 3). Measurement of the 7HE-only group ended at 96 hours as CD19 returned to baseline. (**F**) Comparison of cytotoxicity between enAFP-based SHIFTERS and the 7HE construct, measured 48 hours after initiating coculture with CD19 synNotch EGFR CAR-T cells, which began on day 2 after heat shock, at a 1:1 E:T ratio. (**G**) Experimental timeline for spheroid formation and imaging. Hep3B SHIFTERS cells (20,000) were seeded 1 day before spheroid formation, combined with CD19 synNotch EGFR CAR-T cells (20,000) on day 0, treated with heat shock as indicated, and imaged at 24, 72, 120, and 168 hours (days 1, 3, 5, and 7) after T cell addition. Created in BioRender. Guo, T. (2026) https://BioRender.com/us2hfz9. (**H**) Representative fluorescence images of BFP-expressing Hep3B spheroids at days 1, 3, 5, and 7, comparing non-HS and HS groups. Scale bar, 500 μm. (**I**) Mean fluorescence intensity (MFI) of constitutive marker BFP in spheroids over time under non-HS and HS conditions (*n* = 3). Statistical significance: ns, not significant; **P < 0.01, ***P < 0.001, and ****P < 0.0001. All data are presented as mean ± SD. Two tailed Student’s *t* tests are applied for statistical analysis.

Beyond hypoxia-driven control, SHIFTERS’ modular design allows integration of other tumor-specific cues through promoter substitution. The α-fetoprotein (AFP) gene serves as a hallmark of hepatocellular carcinoma (HCC), which remains transcriptionally silent in adult liver but becomes markedly up-regulated in HCC (*59*). The enhancer-promoter cassette that governs AFP expression, enAFP, exploits AFP’s tumor-restricted regulation to confine transgene expression specifically to HCC cells (*31*, *32*). We hence also examined whether SHIFTERS can be extended to exploit this cancer-intrinsic hallmark for CAR-T therapy by replacing the hypoxia response element with enAFP, ensuring robust activity in HCC cells while minimizing activities in healthy liver tissue (Fig. 4A). This approach allows SHIFTERS to induce CD19 expression in a tumor cell-type-specific manner, adding a biological layer of control beyond the physical spatiotemporal regulation of ultrasound. To confirm the specificity of the enAFP promoter, we tested its activity across four cell lines: Lenti-X 293T (Lenti-X), LNCaP, U251, and Hep3B2.1-7 (Hep3B) (HCC). We observed strong enAFP-driven green fluorescent protein (GFP) expression exclusively in the Hep3B liver cancer cell line (Fig. 4B). We then introduced the enAFP-based SHIFTERS construct into both Hep3B (HCC) and U251 (non-HCC) cell lines and evaluated CD19 expression with or without heat shock. Robust CD19 production was observed only in the HS+ HCC condition (Fig. 4, C and D). Consistent with earlier observations, this enAFP-based SHIFTERS also maintained significantly longer and higher CD19 expression over time than the 7HE-only approach (Fig. 4E), supporting the potential to enhance synNotch CAR-T cell activation through sustained antigen presentation.

We further applied this approach to induce sustained and cell-type-specific antigen expression for cancer eradication by integrating with the synNotch CAR-T–mediated cytotoxicity. SHIFTERS induced a significant tumor cell killing upon heat shock, consistent with the persistent CD19 production kinetics (Fig. 4, E and F). In contrast, the control group with 7HE-CD19 led to less tumor cell killing upon heat shock stimulation (Fig. 4F), reflecting the transient CD19 expression (Fig. 4E). The enAFP-based SHIFTERS Hep3B cells were also introduced into spheroids with or without heat shock stimulation, followed by the coculture with CD19 synNotch CAR-T cells (Fig. 4G). Similarly, treated spheroids showed effective tumor clearance (Fig. 4, H and I, and fig. S6B). These results verified the modular design of SHIFTERS, extending from the hypoxia-responsive element to tumor-specific promoters for precise and potent therapeutics against various solid tumors.

### FUS-controllable SHIFTERS is effective for tumor immunotherapy in vivo

**Fig. 5.**
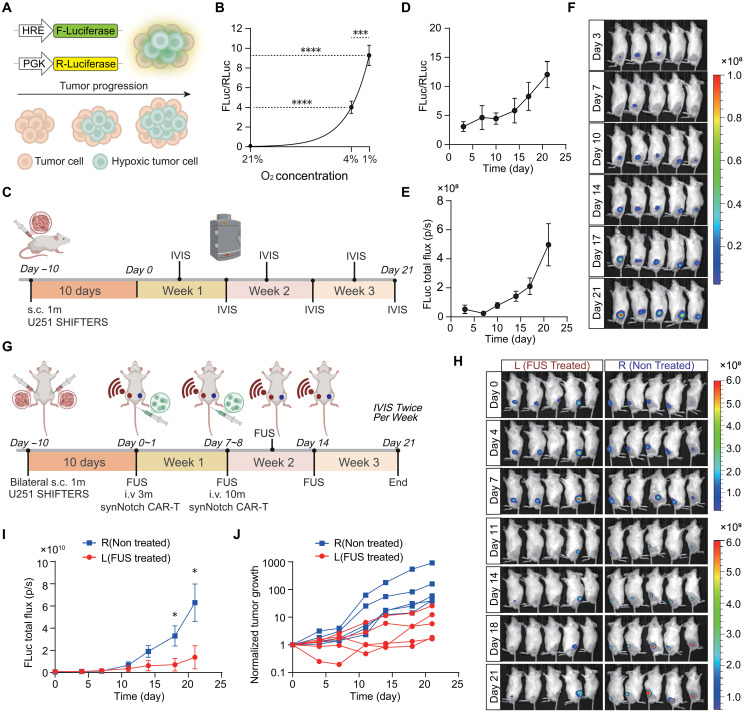
FUS-controllable SHIFTERS is effective for tumor immunotherapy in vivo. (**A**) Schematic of the dual-luciferase reporter system, with an HRE-driven Firefly luciferase (FLuc) and a constitutive RLuc. (**B**) In vitro FLuc/RLuc ratios under varying oxygen concentrations (21, 4, and 1%), two tailed t tests were used to analyze the data, the data are plotted with Mean ± SD (*n* = 4). Exponential growth equation was used to Least-Squares fit the data, with R^2^ = 0.9749. (**C**) Timeline for subcutaneous implantation of U251 cells and longitudinal BLI over 3 weeks. Created in BioRender. Guo, T. (2026) https://BioRender.com/zzst8w6. (**D**) In vivo FLuc/RLuc ratios recorded over time from subcutaneous tumors. Data are presented as mean ± SEM (*n* = 5). (**E**) In vivo total flux of FLuc signal over time. Data are presented as mean ± SEM (*n* = 5). (**F**) Bioluminescence images of mice bearing subcutaneous dual-luciferase reporter tumors at various time points (color scale: min = 2.50 × 10^6^, max = 1.00 × 10^8^, radiance unit = p/sec/cm^2^/sr). (**G**) Experimental timeline for bilateral subcutaneous U251 SHIFTERS tumor implantation, focused-ultrasound (FUS) treatment, and systemic intravenous (i.v.) delivery of CD19 synNotch EGFR CAR-T cells. Created in BioRender. Guo, T. (2026) https://BioRender.com/n21wv70. (**H**) Bioluminescence images at indicated time points, showing bilateral tumors in FUS-treated (L, left) and nontreated (R, right) conditions (color scale; top: min = 2.00 × 10^7^, max = 6.00 × 10^8^; bottom: min = 6.00 × 10^8^, max = 6.00 × 10^9^, radiance unit = p/sec/cm^2^/sr). (**I**) Total flux signal over time for FUS-treated (left) and nontreated (right) tumors. Data are presented as mean ± SEM. One-tailed paired *t* test was used to analyze the data (*n* = 5). (**J**) Normalized tumor growth curves for individual mice, comparing FUS-treated (left) and nontreated (right) tumors, normalizing to the day 0 measurement. Statistical significance: *P < 0.05, ***P < 0.001, and ****P < 0.0001.

We then move forward to test the functions of FUS controllable SHIFTERS in vivo. Subcutaneously injected U251 cells expressing SHIFTERS exhibited exponential tumor growth over time (fig. S7). We then examined Firefly luciferase (FLuc) as a bioluminescent reporter to monitor the hypoxia development in U251 tumors. To account for tumor growth, we coexpressed constitutive Renilla luciferase (RLuc) for tumor size normalization (Fig. 5A). We first calibrated this dual-luciferase reporter in vitro at 21, 4, and 1% O_2_ for 24 hours to establish a reference curve. Under normoxia (21% O_2_), the normalized FLuc/RLuc ratio remained near zero (0.08), indicating minimal background hypoxia signaling. As oxygen levels decreased, the signal rose exponentially (exponential fit *R*^2^ = 0.9749). Between 4 and 1% O_2_, the reporter became highly responsive, with FLuc/RLuc ratio escalating from 4.01 to 9.27, demonstrating its strong sensitivity to hypoxia (Fig. 5B). After these calibrations, 1 million U251 cells expressing the bioluminescence reporter were subcutaneously implanted into NOD scid gamma (NSG) mice, and after a 10-day growth period, we monitored dual-luciferase signals twice weekly to track the tumor growth and hypoxia development (Fig. 5C). This subcutaneous tumor model exhibited a progressively increasing FLuc/RLuc ratio, confirming the emergence of the hypoxic tumor microenvironment (Fig. 5D). The FLuc signal alone also displayed a robust and steadily increasing trajectory, verifying a persisted and expanded hypoxic niche (Fig. 5, E and F), which was supported by the Hypoxyprobe staining of the tumor tissue upon the end of the experiment (fig. S8).

To examine the utility of SHIFTERS for FUS-guided tumor priming in vivo, we established a bilateral flank model in NSG mice by implanting SHIFTERS U251 cells constitutively expressing FLuc on both sides, applying FUS to temporarily induce antigen to only one flank while leaving the contralateral tumor untreated as an internal control. This design ensures that both tumors experience the same host environment and the same systemic exposure to infused T cells. We administered CD19 synNotch CAR-T cells systemically via tail vein following FUS stimulation on the left flank (Fig. 5G). After an initial dose (3 million cells), only two mice showed modest tumor regression in the FUS-treated tumor relative to the untreated contralateral control. We then administered a second, higher dose (10 million cells) together with an additional FUS treatment to maintain antigen induction and sustain synNotch priming within the treated tumor (Fig. 5G). Under these conditions, FUS-treated tumors were substantially suppressed, whereas contralateral untreated tumors continued to progress (Fig. 5, H to J). Collectively, the flank-restricted efficacy observed in this internally controlled model supports the conclusion that SHIFTERS, when coupled to localized FUS stimulation, can promote spatially targeted CAR-T killing and tumor regression. A central feature of synNotch circuits is that effector CAR expression is induced only upon local priming and then wanes after the priming cue dissipates, which is expected to confine maximal cytotoxic activity to the priming site and to limit activity once T cells leave that environment (*60*). The preferential regression of the FUS-treated tumor provides the net consequence of trafficking/retention, priming-dependent effector engagement, and serial cytotoxicity over time of synNotch CAR-T therapy.

## DISCUSSION

In this study, we developed SHIFTERS, a dual-input AND-gate circuit that converts tumor-intrinsic signals into remotely controllable, noninvasive expression of the clinically validated antigen CD19 for safe activation of CD19 synNotch CAR-T cells. The circuit uses a split Gal4-VP64 system, with VP64 controlled by a tumor hallmark input, a hypoxia-responsive promoter (HRE) or a cell-type-specific promoter, and Gal4 controlled by a FUS-inducible heat-shock promoter (7HE), restricting CD19 production to regions with both tumor-specific signals hypoxia and FUS activation. SHIFTERS demonstrated potent antitumor efficacy with synNotch CAR-T cells across 2D cocultures, 3D spheroids, and in vivo models either under hypoxia or in HCC cells harboring the specific enhanced AFP (enAFP) promoter. This modularity supports broader application through tumor-type-specific promoter adaptation and establishment of localized antigen-presenting niches within heterogeneous tumors. Low basal CD19 expression is not predicted to generate substantial synNotch priming relative to fully activated SHIFTERS, particularly in vivo (Fig. 5I and fig. S7B). Specifically, synNotch “prime-and-kill” circuits introduce inherent buffering and thresholding: synNotch-CAR T cells are precisely restricted to tumor region (*60*), and priming must be sustained and sufficiently strong to drive CAR transcription/translation and accumulate functional CAR levels. Weak or transient antigen exposure remains functionally subthreshold, an intentional feature leveraged for antigen-density discrimination (*56*). Moreover, synNotch-induced CAR expression is transient, with an approximately 8-hour half-life after withdrawal of the priming signal, limiting durable effector programming from brief or low-level stimulation (*60*). Last, the functional impact of any basal leakage depends on experimental context (e.g., E:T ratio and exposure duration). Effective tumor killing requires adequate numbers of primed CAR-expressing T cells and sufficient engagement to overcome tumor growth kinetics. Therefore, modest background antigen levels may be detectable by flow cytometry yet remain insufficient to trigger robust synNotch-driven cytotoxic activity. These results establish SHIFTERS as a customizable, tumor-constrained, clinician-controllable platform to improve the safety, precision, and therapeutic durability of CAR-T cell therapy in solid tumors.

SHIFTERS enables the creation of spatially confined training centers by converting a subset of tumor cells into CD19^+^ senders that train synNotch CAR-T cells, which then mediate bystander clearance of adjacent CD19^−^ tumor cells. In 2D cocultures, introducing a small fraction of CD19^+^ cells was sufficient to train the CD19 synNotch CAR-T cells and drive substantial elimination of the overall tumor population, and SHIFTERS-trained CD19 synNotch CAR-T cells efficiently killed wild-type cancer cells. In 3D spheroids, SHIFTERS achieved complete eradication despite heterogeneous hypoxic responsiveness. This exemplifies the synNotch prime-and-kill paradigm, where a limited priming antigen produces potent, localized elimination despite antigen heterogeneity (*60*). Unlike direct cytotoxic/suicide-gene approaches that are constrained by delivery efficiency, this combinatorial strategy exploits a minority of induced tumor cells as training centers to activate synNotch CAR T cells and eradicate the broader tumor mass. Moreover, because synNotch-driven CAR expression is transient and decays as T cells leave the priming zone (*61*), this platform permits highly potent yet less specific CARs, such as EGFR CAR (*62*) used in this study, while mitigating off-tumor toxicity.

The SHIFTERS platform introduces a programmable strategy that converts tumor hallmarks into therapeutic antigens precisely controlled by FUS for synNotch CAR-T therapy. Unlike approaches relying solely on tumor-specific promoters, SHIFTERS incorporates ultrasound-induced hyperthermia, providing refined spatial and temporal antigen control: The FUS system we used has a spatial confinement in millimeters precision (*35*, *63*), thereby restricting heat-shock induction to the insonated focal area and minimizing exposure of surrounding tissues; moreover, because heat-shock signaling is intrinsically transient, ultrasound-induced hyperthermia creates a defined “on-window” in which promoter activity rises following stimulation and then diminishes as tissue temperature returns to baseline, making antigen induction reversible and controllable through re-dosable sonication rather than continuous expression (*35*, *42*). This integration holds promise for reducing off-target effects, particularly in hypoxia-driven targeting. The spatiotemporal regulation offered by ultrasound is crucial, confining CAR-T activation to a well-defined site while minimizing risks of prolonged or excessive toxicity. Apart from advantages granted by FUS, the SHIFTERS design achieves superior antigen production compared to FUS-only systems by using Gal4-UAS transcriptional amplification. Moreover, SHIFTERS further extends antigen duration by integrating sustained tumor-specific signals to the transient heat-shock response, bolstering CAR-T cell activation for a longer period, potentially reducing the required ultrasound treatment frequency. In combination with synNotch CAR-T cells, whose priming logic supports on-site activation, transient effector CAR expression, and reduced exhaustion (*60*, *64*), SHIFTERS integrates (i) tumor-hallmark specificity, (ii) externally programmable FUS spatiotemporal regulation, and (iii) synNotch prime-and-kill behavior to provide a controllable and robust approach to solid-tumor immunotherapy.

In future iterations, SHIFTERS’ modular architecture permits incorporation of additional FUS-gated cytokines and payloads to tumor, alongside CD19, to enhance synNotch CAR-T efficacy while minimizing off-tumor activation, including interleukin-2 (IL-2), IL-18, and/or interferon-γ (*65*–*68*). Cosecretion of such cytokines with CD19 may help synNotch CAR-T cells overcome immunosuppressive tumor microenvironments. We acknowledge the current format of this platform relies on pre-engineered cell lines rather than direct gene cassette delivery into tumors, representing an idealized experimental scenario for establishing circuit logic and biological feasibility. The advancement for SHIFTERS to facilitate clinical translation requires future development of gene-delivery techniques, such as lipid nanoparticles delivering mRNA (*69*) or adeno-associated viruses delivering DNA (*70*). Nevertheless, we have successfully demonstrated the feasibility of leveraging tumor hallmarks combined with FUS to create inducible “kill-me” signals, presenting a potent and safe strategy to address persistent challenges in solid tumor treatment. Moreover, our engineered system exhibits substantial modularity, highlighting its broad applicability and potential as a versatile platform for customized cancer therapies.

## MATERIALS AND METHODS

### Cloning

Plasmid DNA sequence and information is included in table S1. Gene templates of the components used in this work were constructed using polymerase chain reaction (PCR) amplification. PCR was performed using synthesized primers (Integrated DNA Technologies) and Q5 DNA polymerase (NEB, M0491). The amplified gene elements were cloned into the modified pHR lentiviral transfer vector, a gift from Wendell Lim (Addgene plasmid no. 79124) digested using EcoR I and Not I, using Gibson Assembly (NEB, E2611L). Constructs with SunTags are cloned through T4 ligation (NEB, M0202L). The sequences of the constructed plasmids were verified by Sanger sequencing (Genewiz, Azenta Life Sciences).

### General mammalian cell culture and hypoxia culture

U251 cells and Lenti-X cells were cultured in Dulbecco’s modified Eagle’s medium (DMEM) (Gibco, 11995115). Hep3B cells were cultured in Eagle’s minimum essential medium (EMEM) (American Type Culture Collection, 30-2003). LNCaP cells and Jurkat cells were cultured in Roswell Park Memorial Institute (RPMI) 1640 Medium (Gibco, 11875093). All cell culture media were supplemented with 10% fetal bovine serum (FBS) (Gibco, 10438026) and 1% penicillin-streptomycin (P/S) (Gibco, 15140122). All cultures were kept at 37°C in a 5% CO_2_ humidified incubator. Hypoxia culture was established using oxygen concentration controlling incubators (Heracell VIOS 160i Tri-gas CO_2_ incubator, Thermo Fisher Scientific), with 1% O_2_ (or 4 and 10% as stated in each experiment). Cells designated for hypoxia condition were preincubated overnight at 1% O_2_ before the experiment to simulate a developed hypoxic microenvironment.

### Cell transduction

Engineered cancer cell lines were generated through lentiviral transduction. Lentiviruses were produced from Lenti-X 293T cells (Clontech Laboratories, no. 632180) cotransfected with a pHR containing plasmid and the viral packaging plasmids pMD2.G and psPAX2 using the ProFection Mammalian Transfection System (Promega, catalog no. E1200). Viral medium/supernatant was collected 48 hours after transfection, filtered with 0.45-μm filter (Sigma-Millipore), and concentrated using Lenti-X Concentrator (TaKaRa, catalog no. 631232). The virus titer was calculated by transducing 0.2 million Jurkat cells with 2 μl of virus, measuring the fluorescence by flow cytometry and calculating its titer with positive percentage. To generate the cell lines, we added the concentrated virus with multiplicity of infection (MOI) of 1 into the cells, which were seeded with a density of 1 × 10^5^ cells in a tissue cultured six-well plate 1 day before transduction. Cells were sorted by fluorescence-activated cell sorting (Sony, SH800) 3 days post-transduction based on their constitutive fluorescent markers.

### Isolation and transduction of primary human T cells

Peripheral blood mononuclear cells were acquired from the San Diego Blood Bank and isolated from buffy coats using lymphocyte separation medium (Corning, 25-072-CV). Primary human T cells were subsequently purified using a Pan T Cell Isolation Kit (Miltenyi, 130-096-535) and activated with Dynabeads Human T-Expander CD3/CD28 (Thermo Fisher Scientific, 11141D) at a bead-to-cell ratio of 1:1. These T cells were cultured in RPMI 1640 supplemented with recombinant human IL-2 (100 U/ml; PeproTech, 200-02) and 50 μM 2-mercaptoethanol (Gibco, 31350010). Cultures were maintained at 37°C in a humidified incubator containing 5% CO_2_. Following 48 hours of stimulation, cells were transduced with concentrated lentivirus (MOI of 5 per construct) on plates precoated with retronectin(Takara, T100B). Six days after transduction, beads were magnetically removed, and T cells were stained with anti-myc Alexa Fluor 488 (Cell Signaling Technology, 9B11), washed three times with 1× phosphate-buffered saline (PBS), and sorted using a SONY SH800 sorter.

### Antibody staining and flow cytometry

CD19 staining for flow cytometry was performed using Alexa Fluor 647 anti-human CD19 antibody (clone: SJ25C1) according to manufacturers’ protocols (BioLegend 302220). In general, cells were washed one time with 1× PBS and resuspended in 100 μl 1× PBS containing the suggested amounts of antibodies, incubated in the dark at room temperature for suggested durations, and washed three times before flow cytometry analysis (BD Accuri C6). Gating was based on nonengineered cells with the same staining procedure. Flow cytometry data were analyzed using FlowJo software (FlowJo_v10.9.0).

### Cytotoxicity assay

FLuc-expressing target cell lines were generated by lentiviral transduction and purified using a SONY SH800 cell sorter. Unless otherwise specified, all cancer cell lines used in cytotoxicity assays carried FLuc constructs for luminescence-based viability measurements. In wild-type cytotoxicity experiments, FLuc constructs were present only in WT U251 cells, while SHIFTERS-carrying U251 cells lacked the luciferase reporter. For cytotoxicity assays, 1 × 10^4^ cancer cells were seeded per well in 100 μl culture media in 96-well plates and allowed to adhere for 3 hours (or 48 hours for delayed day 2 cocultures), after which media was replaced with 100 μl RPMI medium containing IL-2 and 2-mercaptoethanol, followed by addition of CD19 synNotch EGFR CAR-T cells at specified E:T ratios relative to the initial 1 × 10^4^ cancer cells. Target cancer cells cultured alone served as controls (“target cancer cell only” control). In training center experiments, the total cell count was maintained at 1 × 10^4^ U251 cells per well, consisting of defined mixtures of CD19^+^/SHIFTERS cells and wild-type cells at specified ratios. Each ratio group included corresponding target cancer cell only controls without T cell coculture. Following incubation for specified durations, luminescence was measured using the One-Glo Luciferase Assay kit (Promega, E6110), and cytotoxicity was calculated as the percentage reduction in luminescence relative to target-only controls using the formula: (1 − luminescence of *X*/luminescence of “target cell only”) × 100%, *X* is the sample measurement.

### 3D spheroid construction

U251 or Hep3B cells containing target genetic constructs were used in the 3D tumor spheroid forming process following published literature (*57*, *58*). In brief, a magnetic matrix (Celerite Labs, Canada) was assembled with corresponding μ-Plate 96 Well Square plates (ibidi, 89621) to generate the uniform tumor spheroids formation. Subsequently, a cell suspension in DMEM with 10% FBS and 1% P/S, supplemented with 25 mM Gadovist, the Gadovist reagent was generously given by R. Sahu (Director of Celerite Labs) was added to each well. The entire system was then incubated at 37°C overnight to facilitate spheroid formation. Gadovist reagent was maintained in the cell culture medium for sustained spheroid intactness.

### 3D spheroid imaging and analysis

Images and related analysis of hypoxia profiles in the tumor spheroids were generated using Incucyte S3 Live-Cell Analysis Instrument. The experimental schedule of 3D spheroid coculture killing is shown in Figs. 3 and 4. Images were acquired using an ECLIPSE-Ti2 microscope (Nikon) and imaging data analysis was conducted with ImageJ. Size was determined using imaging analysis script cytomata (now called fluoressential) from microscopy image, deposited in https://github.com/phuongho43/fluoressential. Analysis parameters were included in table S2. Infiltration analysis was conducted and snapshotted using MICA Microhub (Leica, Germany), and imaging data were analyzed by Imaris 10.2 imaging processing software. SynNotch CAR T cells and tumor cells were identified during 3D reconstruction and cell type identification according to their sizes and internal fluorescence marker (CD19 synNotch CAR T cells: mCherry, SHIFTERS U251 Tumor cells: mCitrine).

### In vitro heat shock

U251/Hep3B cells were pelleted and resuspended in complete DMEM/EMEM at a concentration of 10 to 20 million cells/ml (approximately 0.5 to 1 million cells per 50 μl). The cell suspension was then aliquoted into PCR 8-strip tubes (50 μl each). These tubes were prewarmed at 37°C for 2 min in a PCR thermocycler (Bio-Rad, 1851148) and subsequently incubated at 43°C for 15 min. For spheroid heat shock treatment, the plates containing spheroids were incubated in a water bath at 43°C for 15 min.

### Animals

All animal experiments were conducted with approval from the Institutional Animal Care and Use Committee at the University of Southern California under Protocol 21479, following institutional guidelines and ethical regulations for animal research. Male NOD.CG-Prkdc^scid^ Il2rg^tm1Wjl^/SzJ (NSG) mice (6 to 8 weeks old) were purchased from The Jackson Laboratory.

### In vivo bioluminescence imaging

In this study, tumor mass was monitored twice weekly via bioluminescence imaging (BLI) following established protocols (*42*). BLI was performed using an IVIS Lumina LT Series III imaging system (PerkinElmer). For FLuc detection, mice received an intraperitoneal injection of d-luciferin substrate (150 mg/kg; GoldBio, LUCK-1G), and imaging began approximately 10 min later to capture peak luminescence. For RLuc detection, mice were injected intraperitoneally with coelenterazine substrate (5 mg/kg, GoldBio, CZ25), and imaging commenced approximately 15 min postinjection, continuing until peak signal intensity was reached. When imaging both FLuc and RLuc signals in the same mouse, an 8-hour interval was maintained between imaging sessions to prevent signal overlap. Image analysis was performed using Living Image software (PerkinElmer). Tumor sizes were quantified by measuring the integrated luminescence intensity within a fixed region of interest covering the same tumor area across different time points.

### Immunofluorescence for hypoxia detection

To detect tumor hypoxia, mice were injected intraperitoneally with Solid Pimonidazole HCl (Hypoxyprobe-1) reconstituted in 1× PBS for a 60 mg/kg concentration. After 90 min, tumors were excised, fixed overnight in 4% paraformaldehyde, cryoprotected in 15 and 30% sucrose, embedded in OCT, and frozen at −80°C. Eight- to 10-μm sections were cut, blocked in 2% bovine serum albumin buffer, and incubated overnight at 4°C with anti-pimonidazole, fluorescein isothiocyanate (FITC)–conjugated IgG1 rat monoclonal antibody (clone 11.23.22.R) (1:50). After washing, slides were counterstained with 4′,6-diamidino-2-phenylindole, mounted in antifade medium, sealed, and stored at 4°C. Imaging was performed using a fluorescence microscope with a FITC filter to visualize hypoxic regions at Optical Imaging Facility in Broad Center for Regenerative Medicine & Stem Cell Research, University of Southern California.

### FUS system

We developed a FUS system featuring real-time temperature-control feedback specifically designed for hyperthermia experiments. This setup uses a customized single-element transducer with central frequency of 1.1 MHz, fabricated by a ring-typed lead-zirconate-titanate element (diameter, 70 mm; focal radius of curvature, 65 mm; DL-47, Del Piezo Specialties) with a central aperture measuring 20 mm. A coupling cone (length, 65 mm) was attached to the transducer, featuring a 4-mm-diameter tip opening. This cone, filled with degassed deionized water as the acoustic coupling medium, served to precisely propagate the ultrasound beam. The tip opening of the cone was sealed using an acoustically transparent thin film (Chemplex, 100). The transducer was driven by using a function generator (Stanford Research Systems, SG386) coupled with a 50-dB power amplifier (E&I, 325LA) to transmit the pulsed sine-wave signals.

### FUS stimulation in vivo

Unless otherwise specified, FUS stimulation was applied weekly according to CD19 kinetics determined in vitro. Before FUS stimulation, the flank region of NSG mice designated for treatment was shaved. Anesthesia was induced using a 2% isoflurane-oxygen mixture and maintained at 1.5% throughout the 15-min FUS procedure. To ensure stable body temperature, anesthetized mice were placed on a heating plate (Auber Instruments, WSD-30B) set to 37°C. A needle-type thermocouple was inserted subcutaneously into the tumor site for real-time temperature monitoring. A thin layer of SCAN ultrasound gel (Parker Labs) was applied over the targeted area, and the FUS transducer was carefully positioned above the tumor on the hindlimb, maintaining gentle contact with the gel. The transducer’s coupling cone opening was precisely aligned, ensuring its center matched the tumor’s center.

Real-time temperature data acquired by the thermocouple were integrated into a proportional-integral-derivative (PID) controller, enabling dynamic adjustments of the function generator’s output power to sustain the focal temperature at 43°C for the full 15-min treatment duration. The PID controller and associated device interface code are accessible at: https://github.com/phuongho43/ultrasound_pid.

### Statistical analyses

Statistical analyses were conducted using GraphPad Prism and Microsoft Excel, and the specifics for each figure are provided in the respective figure legends. *t* tests or analysis of variance (ANOVA) were performed to analyze the data as described in each figure legends. Detailed data and statistics to reconstruct each figure can be found at table S2.
