# Supplementary material for: Ultrasound priming gated by solid tumor hallmarks to guide CAR-T therapy
Source: Sci Adv. 2026 Jun 10;12(24):eaed0666. doi: 10.1126/sciadv.aed0666 (PMC13251842; doi:10.1126/sciadv.aed0666)
Supplement: Supplementary file 1 — Figs. S1 to S8 Legends for tables S1 and S2 Legend for movie S1 [file sciadv.aed0666_sm.pdf]

Science Advances
AAAS

Supplementary Materials for

Ultrasound priming gated by solid tumor hallmarks to guide CAR-T therapy

Tianze Guo *et al.*

Corresponding author: Longwei Liu, longweil@usc.edu; Keyue Shen, keyue.shen@usc.edu; Yingxiao Wang, ywang283@usc.edu

Sci. Adv. **12**, eaed0666 (2026)
DOI: 10.1126/sciadv.aed0666

The PDF file includes:

Figs. S1 to S8
Legends for tables S1 and S2
Legend for movie S1

Other Supplementary Material for this manuscript includes the following:

Tables S1 and S2
Movie S1

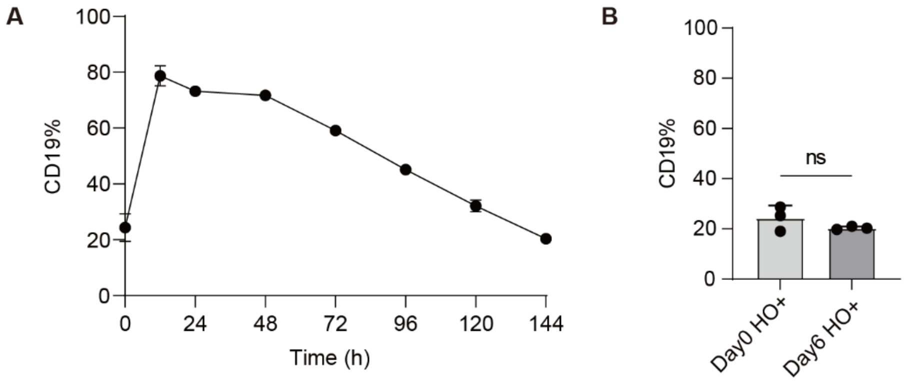

Fig. S1. Temporal reversibility of SHIFTERS-induced CD19 expression

(A) Full time-course quantification of the percentage of CD19-positive (CD19%) cells following induction for 6 days, appending from the data shown in Fig. 1K. Data are presented as mean ± standard deviation. **(B)** Comparison of CD19% between Day 0 prior to heat shock (HS) activation and Day 6 post HS activation, under hypoxia condition. No significant difference was detected (ns, not significant. n=3). Two-tailed Student's t test is used to analyze the data. Indicated p value is 0.2381.

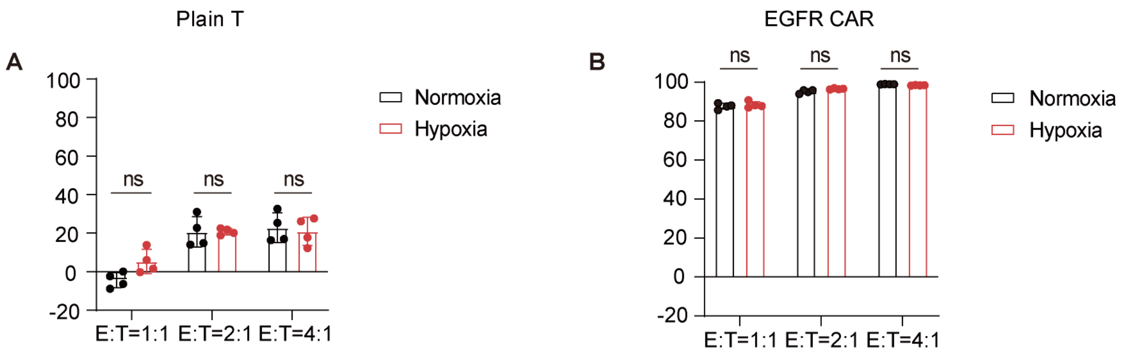

Fig. S2. Hypoxia does not impact CAR-T killing in vitro

(A) Cytotoxicity of plain T cells at different E:T ratios under normoxia (21% O2) and hypoxia (1% O2). **(B)** Cytotoxicity of constitutive EGFR CAR T cells at different E:T ratios under normoxia and hypoxia. Data are presented as mean ± SD. Two-way ANOVA is used to analyze the data. **Statistical significance:** ns, not significant. n = 4.

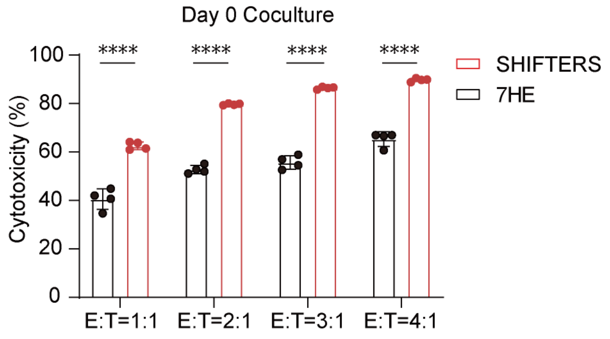

Fig. S3. SHIFTERS shows enhanced cytotoxicity compared to 7HE at day 0

Comparison of cytotoxicity elicited by SHIFTERS and 7HE constructs at various E:T ratios 48h post co-culture on day 0. Note the SHIFTERS U251 was cultured constantly under hypoxia conditions whereas 7HE group was staying at normoxic throughout coculture. Two tailed Student's t tests are used to analyze the data. **Statistical significance:** ****p < 0.0001

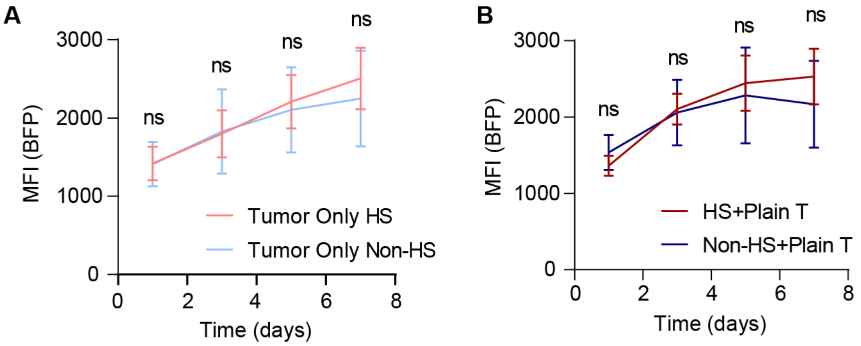

Fig. S4. U251 SHIFTERS spheroid will not be affected by heat shock and plain T cells

(A) and (B) Mean fluorescence intensity (MFI) of constitutive marker BFP in spheroids over time under Non-HS and HS conditions. U251 SHIFTERS cells (20,000) are plated one day prior to spheroid formation, then combined with plain T cells (20k) on Day 0 (B). Heat shock (HS) treatment is applied as indicated, and spheroids are imaged at 24h, 72h, 120h, 168h. data analyzed using microscopy images using ImageJ. Two tailed Student's t tests are applied for statistical analysis (n=4). **Statistical significance:** ns, not significant.

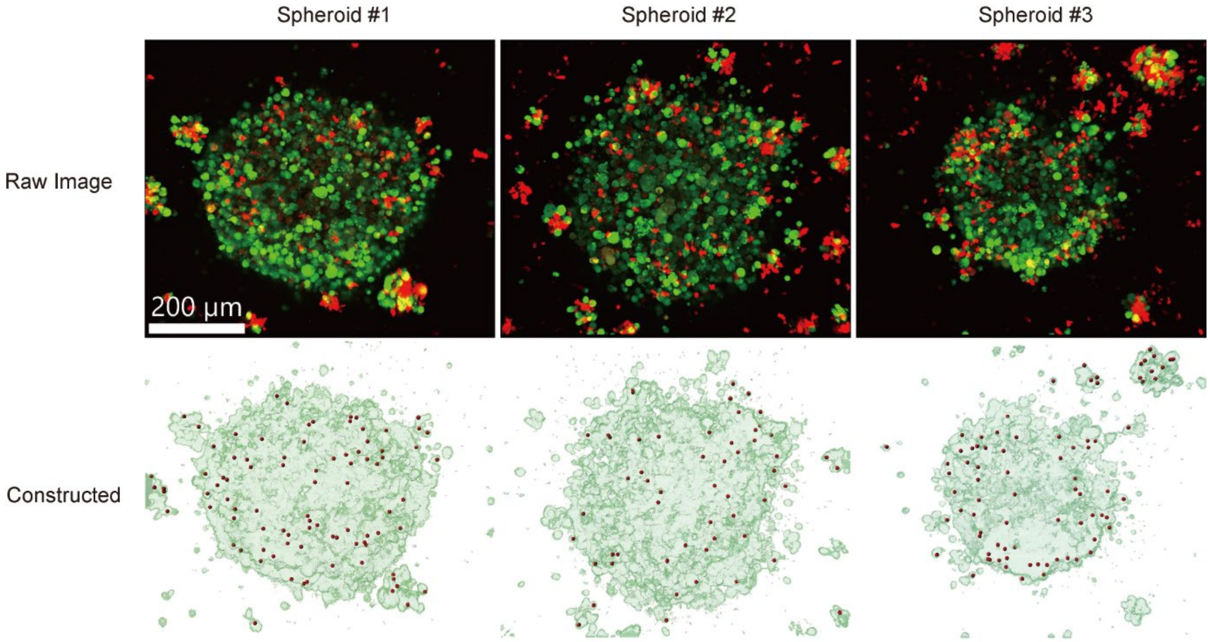

Fig. S5. Confocal imaging demonstrates intratumoral infiltration of synNotch CAR-T cells into heat-shock–activated U251 SHIFTERS spheroids

Representative confocal images of three independent U251 tumor spheroids (Spheroids #1–#3) following heat-shock activation and coculture with CD19 synNotch EGFR CAR-T cells (E:T=1:1, cell number=20,000), imaged at 24 h post-coculture. Top row (Raw Image): merged confocal images showing tumor spheroids (green) and CAR-T cells (red). Bottom row (Constructed): reconstructed spatial maps from the z-stack highlighting spheroid boundaries (light green outlines) and T-cell positions (red dots), only infiltrating T cells (within constructed boundary) are indicated. 200 µm scale bar is shown at top left and is consistent among the groups.

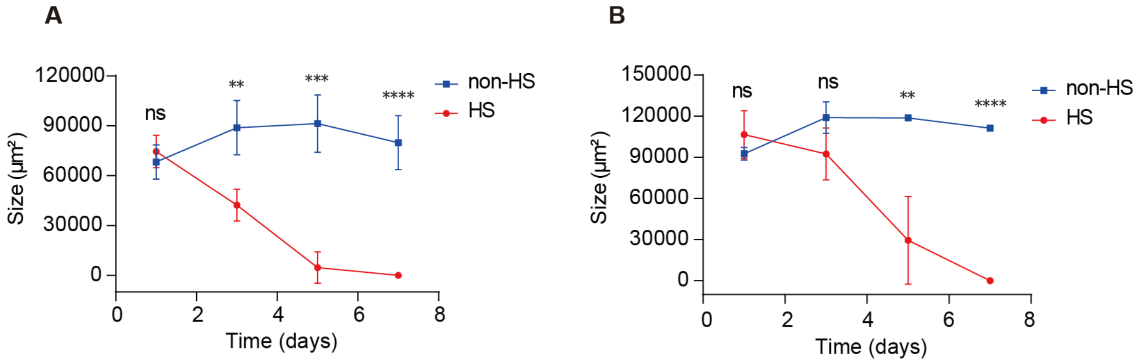

Fig. S6. Complementary size analysis confirmed effective tumor suppression in 3D spheroid models.

(A) and (B) Size analysis of spheroids shown in Fig. 3E (A) and Fig. 4I (B) over time under non-HS and HS conditions, size was determined using imaging analysis script cytomata from the same microscopy image used for Fig. 3E and Fig. 4I. Data is presented as mean ± SD. Two tailed t tests are applied for statistical analysis (n=4). **Statistical significance:** ns, not significant; $^{**}p < 0.01$; $^{***}p < 0.001$; $^{****}p < 0.0001$.

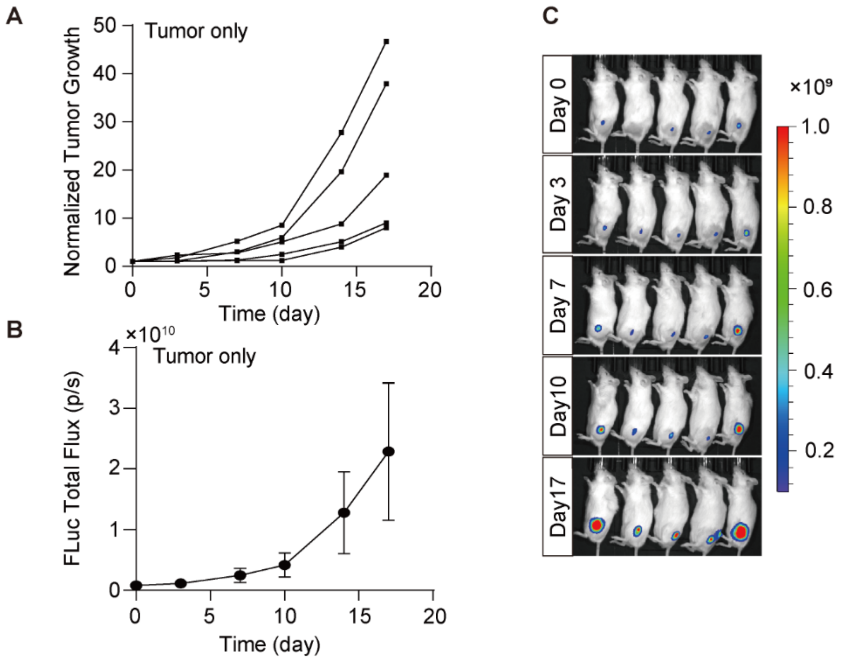

Fig. S7. Subcutaneous SHIFTERS U251 model can expand naturally

(A) Normalized tumor growth curves for subcutaneous SHIFTERS U251 tumors grown without any treatment (n=5). **(B)** Total Flux signal over time from BLI image. **(C)** Representative Bioluminescence images of tumor-bearing mice over time, illustrating changes in tumor-derived luminescence signals (Color Scale: Min=1.00e8, Max=1.00e9, Radiance Unit=p/sec/cm2/sr).

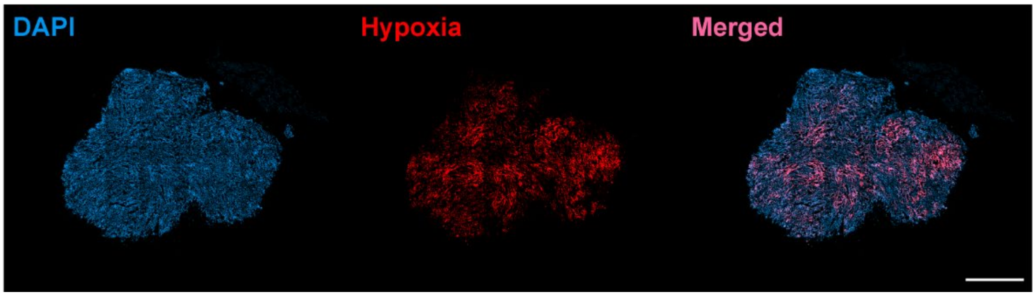

Fig. S8. Hypoxia in tumor tissues validated by hypoxia staining

Representative immunofluorescence image showing hypoxic niche within a section of tumor tissue. Tumor sections were stained with DAPI (blue) to visualize nuclei and Hypoxyprobe^{TM} (red) to detect hypoxic regions. The merged image illustrates co-localization of hypoxic areas within the tumor spheroids. Scale bar represents 1000 µm.

Movie S1. Tumor spheroid model develops hypoxia over time
Incucyte time-lapse image of 9,000 U251 hypoxia reporter cells (used in Figure 3B) in an hourly interval for 24 hours, scale bars shown in the bottom left. Each frame represents one hour. UnaG expression is obtained with a standard GFP filter, brightfield images and fluorescence images were stacked using ImageJ.

Table S1.
Plasmid sequences of the gene constructs used in this work

Table S2.
Data file that used to plot each corresponding figures in text with relevant statistics
